# Transcranial Magnetic Stimulation to the Occipital Place Area Biases Gaze During Scene Viewing

**DOI:** 10.3389/fnhum.2018.00189

**Published:** 2018-05-08

**Authors:** George L. Malcolm, Edward H. Silson, Jennifer R. Henry, Chris I. Baker

**Affiliations:** ^1^School of Psychology, University of East Anglia, Norwich, United Kingdom; ^2^Laboratory of Brain and Cognition, National Institute of Mental Health, National Institutes of Health, Bethesda, MD, United States

**Keywords:** occipital place area, gaze control, transcranial magnetic stimulation (TMS), scene understanding, visual fields

## Abstract

We can understand viewed scenes and extract task-relevant information within a few hundred milliseconds. This process is generally supported by three cortical regions that show selectivity for scene images: parahippocampal place area (PPA), medial place area (MPA) and occipital place area (OPA). Prior studies have focused on the visual information each region is responsive to, usually within the context of recognition or navigation. Here, we move beyond these tasks to investigate gaze allocation during scene viewing. Eye movements rely on a scene’s visual representation to direct saccades, and thus foveal vision. In particular, we focus on the contribution of OPA, which is: (i) located in occipito-parietal cortex, likely feeding information into parts of the dorsal pathway critical for eye movements; and (ii) contains strong retinotopic representations of the contralateral visual field. Participants viewed scene images for 1034 ms while their eye movements were recorded. On half of the trials, a 500 ms train of five transcranial magnetic stimulation (TMS) pulses was applied to the participant’s cortex, starting at scene onset. TMS was applied to the right hemisphere over either OPA or the occipital face area (OFA), which also exhibits a contralateral visual field bias but shows selectivity for face stimuli. Participants generally made an overall left-to-right, top-to-bottom pattern of eye movements across all conditions. When TMS was applied to OPA, there was an increased saccade latency for eye movements toward the contralateral relative to the ipsilateral visual field after the final TMS pulse (400 ms). Additionally, TMS to the OPA biased fixation positions away from the contralateral side of the scene compared to the control condition, while the OFA group showed no such effect. There was no effect on horizontal saccade amplitudes. These combined results suggest that OPA might serve to represent local scene information that can then be utilized by visuomotor control networks to guide gaze allocation in natural scenes.

## Introduction

Understanding our visual world is an active process. As we move and interact with our environment, we continuously convert sensory cues from light reflecting off surfaces into semantically meaningful information. This information is then exploited to complete a range of goals including categorization (e.g., city street), search (find a road sign), navigation (take a left at the upcoming traffic lights), action affordances (slow down at light) and more (Malcolm et al., 2016). The ability to parse and understand visual input facilitates our ability to interact efficiently with the surrounding environment.

There are three cortical regions that exhibit preferential responses to viewed scene images over other high-level stimuli such as faces and objects: the parahippocampal place area (PPA Epstein and Kanwisher, 1998) on the ventral temporal surface; the medial place area (MPA; also referred to as retrosplenial complex, RSC; Silson et al., 2016b) on the medial parietal surface, and the occipital place area (OPA; also referred to as transverse occipital sulcus, TOS; Dilks et al., 2013) on the lateral occipital surface (for review, see Epstein, 2014). Of the three, OPA has received comparatively less research focus, leaving its role within the scene processing network unclear in terms of the visual properties it responds to as well as its overall function. In this study, we investigate the potential role of OPA in providing scene information for the guidance of eye movements.

The role of OPA in scene understanding has often been considered in relation to that of PPA. Previous proposals have noted that OPA’s relative posterior position to PPA is similar to the posterior-anterior arrangement of cortical regions within the face processing network (occipital face area (OFA), and the fusiform face area (FFA), respectively) and the body processing network (extrastriate body area (EBA), and the fusiform body area (FBA), respectively), both of which demonstrate a local-to-global stimulus hierarchy of visual information (Taylor et al., 2007). In that vein, Kamps et al. (2016a) reported that OPA responds to local scene regions while PPA shows greater sensitivity to global scene properties. However, more recent evidence suggests that the redundancy of scene selective regions across both lateral and ventral surfaces of occipitotemporal cortex reflect differential biases for the lower and upper contralateral visual fields, respectively (Silson et al., 2015). As these cortical regions are responsive to differing segments of the visual field, it is unlikely that the lateral and ventral surface are stages within a visual representation hierarchy. Instead, they may reflect duplicated selectivity for different portions of the visual field and be associated with distinct and separable functions. For example, the upper visual field is likely to contain large-scale objects such as buildings and landmarks that are important for navigation, while the lower visual field is more likely to contain objects (Greene, 2013) and may be more relevant for movement of the body through space (Malcolm et al., 2016). Indeed, while PPA exhibits strong responses to navigationally-relevant objects (Auger et al., 2012; Troiani et al., 2014), recent data suggests that OPA may be involved in representing navigational affordances such as the presence of boundaries (Julian et al., 2016) or paths (Bonner and Epstein, 2017).

In this context it is important to note that OPA’s occipito-parietal location likely feeds information into parts of the dorsal pathway critical for eye movements (Kravitz et al., 2011). OPA’s role in scene understanding may therefore also include acquiring environmental information for the purpose of directing gaze to critical parts of the scene (generally objects, Henderson and Hollingworth, 1998; Xu et al., 2014; Malcolm and Shomstein, 2015) for foveal processing.

In the present study, we investigate if OPA is causally involved in gaze allocation during scene viewing by utilizing transcranial magnetic stimulation (TMS) during a free-viewing, eye movement experiment. Previous studies applying TMS to OPA have focused more on global properties, such as demonstrating that the region is causally involved in scene perception (Dilks et al., 2013), contributes to superordinate natural/non-natural category judgments of scenes, but not objects (Ganaden et al., 2013), and potentially plays a role in navigation by representing boundaries or paths during navigation tasks (Julian et al., 2016; Bonner and Epstein, 2017). Here, we take advantage of the regions’ strong retinotopic representations, predominantly of the contralateral lower visual field (Silson et al., 2015), and hypothesize that interference in this cortical region would manifest itself in disrupted gaze allocation into the contralateral, and potentially lower, visual field. TMS was applied over right OPA (rOPA) while participants viewed scene images. If OPA plays a causal role in the guidance of eye movements within scenes, then TMS to rOPA, but not a control site with a similar contralateral and lower visual field preference (right occipital face area, rOFA; Silson et al., 2016a), should disrupt gaze allocation. Target sites for TMS were the peak voxel of scene selectivity within rOPA and face selectivity within rOFA, respectively. In particular, given the strong contralateral representation within rOPA, we predicted that participants should be biased for making eye movements toward the ipsilateral visual field (here, right visual field) and inhibited to the contralateral visual field (left visual field), following stimulation to rOPA, but not rOFA, with potentially additional effects for the lower over upper visual field.

## Materials and Methods

### Participants

Twenty-four participants were recruited (16 female, mean age = 23.5 years ± 2.2), half of whom were assigned to the OPA group (8 female, mean age = 22.4 years ± 1.2) and half to the OFA group (8 female, mean age = 24.5 years ± 1.7). All participants had normal or corrected-to-normal vision and provided informed and written consent in accordance with the declaration of Helsinki. The National Institutes of Health Institutional Review Board approved the consent and protocol. This work was supported by the Intramural Research program of the National Institutes of Health—National Institute of Mental Health Clinical Study Protocols 93-M-0170 (NCT00001360), 12-M-0128 (NCT01617408).

### fMRI Scanning Parameters

Participants were scanned on either a 3.0T GE Sigma MRI scanner (Scanner 1) or a 3.0T GE 750 MRI scanner (Scanner 2) in the Clinical Research Center on the National Institutes of Health campus (Bethesda, MD, USA) as part of independent experiments.

Across scanners, partial volumes of the occipital and temporal cortices were acquired.

**Scanner 1**: eight-channel head coil; 28 slices; 3 mm^3^ voxel size with 10% interslice gap; TR = 2. TE = 30 ms; matrix size = 64 × 64, FOV = 192 mm.

**Scanner 2**: thirty-two channel head coil; 37 slices; 3 mm^3^ voxel size with 10% interslice gap; TR = 2. TE = 30 ms; matrix size = 64 × 64, FOV = 192 mm.

### Anatomical Scanning Parameters

T1-weighted anatomical scans were acquired for each participant using the magnetization-prepared rapid gradient echo (MPRAGE) sequence (TR = 2080 ms, TE = 2.43 ms, flip angle = 9°, voxel size 1 mm^3^, matrix size 256 × 256, FOV = 256 mm).

### fMRI Visual Stimuli and Tasks

We conducted two separate fMRI experiments: category-selective functional localizers to identify the target sites for TMS, and population receptive field (pRF) mapping to determine the visual field representations within the targeted regions.

### Category-Selective Functional Localizers

All participants completed six runs in order to localize scene and face selective areas of occipitotemporal cortex. In these runs, images from six categories (5 × 5°) including scenes, faces, buildings, bodies, objects and scrambled objects, were presented in blocks (16 s) separated by fixation periods (8 s). There were 20 stimuli per block (300 ms per stimulus followed by 500 ms fixation). Each run contained 12 stimulus blocks, with each category occurring twice per run in a counterbalanced order. Participants fixated a central cross and performed a one-back task throughout each run, indicating via button-press every time the same image was repeated sequentially. The total length of each run was 256 s.

### Population Receptive Field Mapping

During pRF mapping runs, a bar aperture traversed gradually through the visual field, whilst revealing randomly selected scene fragments from a total of 90 color images. During each 36 s sweep the aperture took 18 evenly spaced steps every 2 s (1TR) to traverse the entire screen. During each bar position five scene fragments were displayed in rapid succession (400 ms per image). Across the 18 aperture positions all 90 possible scene images were displayed once. A total of eight sweeps were made during each run (four orientations, two directions). Specifically, the bar aperture progressed in the following order for all (eight) runs: Left—Right, Bottom Right—Top Left, Top—Bottom, Bottom Left—Top Right, Right—Left, Top Left—Bottom Right, Bottom —Top, and Top Right—Bottom Left. The bar stimuli covered a circular aperture (20° diameter). Participants performed a color detection task at fixation, indicating via button press when the white fixation dot changed to red. Color fixation changes occurred semi-randomly, with approximately two-color changes per sweep (Silson et al., 2015). The total length of each run was 288 s.

### fMRI Data Preprocessing

All data were analyzed using the Analysis of Functional NeuroImages (AFNI) software package (Cox, 1996)^1^. Prior to localizer and pRF analyses, all images for each participant were motion corrected to the first volume of the first run, after removal of the appropriate “dummy” volumes (eight) to allow stabilization of the magnetic field. Post motion-correction data were smoothed with a 2 mm full-width at half-maximum Gaussian kernel for the localizer data only.

### Localizer Analysis

To identify scene and face selective regions of interest (ROI), we conducted a standard general linear model implemented in AFNI. Specifically, a response model was built by convolving a standard gamma function with a 16 s square wave for each condition and compared against the activation time courses using Generalized Least Squares (GLSQ) regression. Motion parameters and four polynomials accounting for slow drifts were included as regressors of no interest. To derive the response magnitude per category, *t*-tests were performed between the category-specific beta estimates and baseline. Scene and face selective regions were defined using the statistical contrast of Scenes > Faces (*p* < 0.0001, uncorrected).

### TMS Site Localization

TMS target sites were identified on an individual participant basis using the Brainsight TMS-MRI co-registration system (Rogue Research). In each participant, the results of the statistical contrast of Scenes > Faces were overlaid onto a high-resolution anatomical scan. The rOPA target site was defined as the peak voxel of scene selectivity within the rOPA, with the rOFA target site representing the peak voxel of face selectivity within the rOFA. We chose OFA as the active control site since OPA and OFA both contain retinotopic representations of the contralateral lower visual field but differ in their category selectivity (scene-selective vs. face-selective). Prior TMS studies of OPA have also used OFA as an active control site (e.g., Dilks et al., 2013).

### TMS Stimulation

A Super Rapid2 Magstim stimulator (Magstim, Wales, UK) delivered TMS through a figure-eight coil with a central diameter of 50 mm. On half of the trials, participants received repetitive TMS: a five-pulse train over 500 ms (10 Hz) at 60% of maximum stimulator output, starting at stimulus onset. Pulse-train initiation was controlled by Experiment Builder (SR Research, Canada) which sent TTL signals to the Magstim using a USB-1208HS box (Measurement Computing, USA).

### Eye Tracking

Eye movements were recorded with an EyeLink II (SR-Research, Canada) sampling at a rate of 500 Hz. Viewing was binocular, but only the eye providing the more accurate calibration was used. As the EyeLink II has a headband designed to keep the camera position stable, but which would also overlap with the targeted scalp locations, we stabilized participants’ heads in a chin rest 80 cm from the display, while the eye tracker was fastened to the chinrest’s stand. The two cameras and their stalks were rotated and repositioned so that they faced participants’ eyes.

Collected eye movement data were segmented into temporal and spatial components: temporal analyses looked at saccade latencies (fixation durations), while spatial analyses were split into retinotopic (saccade amplitude) and spatiotopic (fixation position on the stimuli) measures. Eye movement data were analyzed separately depending on whether the following saccade was horizontal or vertical (determined by whether the saccade change in x-coordinates exceeded the change in y-coordinates, or vice-versa) since saccade amplitudes and latencies vary as a function of their direction during free viewing (Tatler and Vincent, 2008). Additionally, data were segmented into two epochs. The first was for fixations starting between 0–400 ms from scene onset, covering fixations that occurred, at least in part, during the TMS pulses. The second was for fixation starting from 401 ms to 1034 ms after scene onset, covering all eye movements after the final TMS pulse.

Experimental sessions were conducted on a Mac G5 computer running OSX Yosemite (10.10.4). Stimuli were shown on a Samsung Syncmaster 244T LCD monitor, with a resolution of 1024 × 640 pixels, and a refresh rate of 60 Hz. Responses were made on a keyboard. Stimulus presentation and response recording was controlled by Experiment Builder (SR Research, Canada).

### Behavioral Visual Stimuli and Task

The behavioral task employed during the TMS portion of the study was primarily intended to encourage participants to make self-initated eye movements across realistic visual scenes to extract information about the objects present.

Participants viewed 128 images of real-world scenes consisting of indoor and outdoor settings, 800 × 600 pixels in resolution, centrally positioned on the monitor. Scenes were selected from Google Images, modified with Adobe Photoshop CS (Adobe, San Jose, CA, USA), and did not contain humans, animals or text. Half of these scenes were assigned to the TMS condition—a TMS pulse train would be provided at their onset—and half were designated control scenes (no TMS pulse). Each scene contained at least three prominent objects, with at least one on each side of the vertical meridian and one on each side of the horizontal meridian. As objects are preferentially fixated over backgrounds (Xu et al., 2014), this positional-distribution encouraged participants to make saccades throughout the scene images.

**Figure 1:**
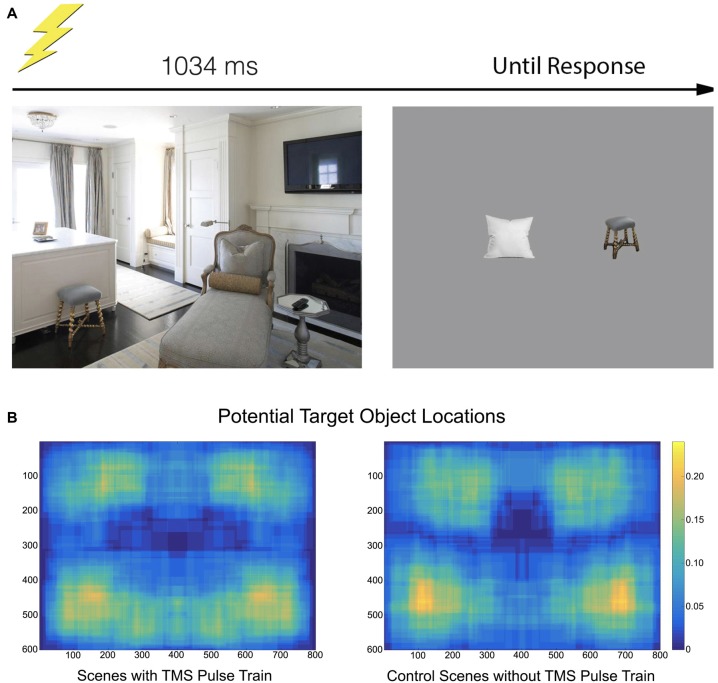
**(A)** The paradigm consisted of a scene appearing for 1034 ms. On half of the trials, a transcranial magnetic stimulation (TMS) pulse train containing five pulses at a rate of 10 Hz was initiated at onset. Participants moved their gaze freely through the scene. After offset, two objects appeared and participants indicated which had been in the previous scene. **(B)** A heatmap indicating where objects specifically used in the recall test were located. The heatmap is drawn from the tightest fitting box around each object, collapsed across scene.

Two objects from each scene were designated potential target objects, one in the upper-half of the scene and one in the lower-half. Some target objects spanned across a midline, but the center of the object was determined to be on one side or the other (Figure 1). Scenes were either presented in their original state or horizontally flipped, counterbalanced across observers, so as to minimize any left-right object position biases. Participants therefore always had something to fixate in the left- and right-side of the image, as well as the upper- and lower-side of the image. Additionally, the position of the designated target object was counterbalanced across all four visual quadrants.

### Procedure

Before the experiment began, each participant underwent the EyeLink 9-point calibration procedure. The eye providing more accurate spatial coordinates was selected for tracking. Re-calibration was performed as necessary during the experiment. Each trial began with a drift correction dot in the middle of the screen. When this was fixated, the experimenter initiated the trial and a scene image would appear for 1034 ms. This was followed by a 1 s blank screen with a fixation cross, and then two objects (one of the target objects and a distractor) presented side-by-side. Participants indicated which of the two objects had been in the previous scene (Figure 1). Each scene had two distractor objects associated with it. These were objects that did not appear in the scenes, but were semantically appropriate to the scene category (e.g., a bedside lamp in a bedroom, a cushion in a living room). All target and distractor objects were presented with their size normalized to ~100 pixels in height so that participants could not deduce whether an object appeared based on this dimension alone.

On half of the trials, participants received repetitive TMS at scene onset: a five-pulse train over 500 ms (10 Hz) at 60% of max output. A pulse was therefore sent at 0, 100, 200, 300 and 400 ms. Depending on their assigned group, participants received TMS to either rOPA or rOFA. On the other half of the trials, no pulse was given. Pulse and no pulse trials were randomly intermixed across an experiment, and participants were given a rest break every 16 trials.

## Results

**Figure 2:**
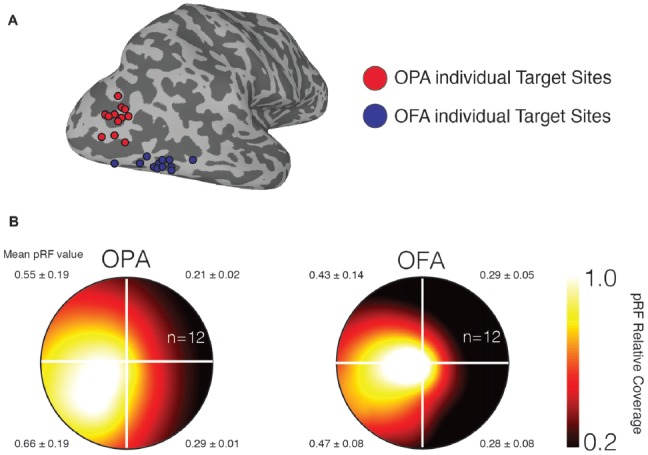
Individual participant TMS target sites and visual field representations. **(A)** Individual participant target sites are shown as 3 mm diameter spheres overlaid onto a surface reconstruction of the right-hemisphere of a representative participant (gyri are light gray, sulci are dark gray). Occipital place area (OPA) target sites are shown in red and are located dorsal of occipital face area (OFA) target sites (blue). **(B)** Group average visual field coverage plots are shown for both target sites. These plots were derived from a 10-mm diameter sphere centered on the peak voxel in each region. Both regions exhibit a strong representation of the contralateral (left) visual field and a smaller bias for the lower visual field. The mean and standard deviation in population receptive field (pRF) value is reported for each quadrant.

To confirm that our stimulation sites demonstrate the retinotopic biases previously reported for OPA and OFA (Silson et al., 2015, 2016a), we computed the average visual field coverage for a sphere (10 mm diameter) centered on the stimulation sites. As expected both stimulation sites showed a strong contralateral bias, with a weaker bias for the upper over lower visual field (Figure 2).

All 24 participants’ data were analyzed. The experimental software crashed after one OPA participant had completed 121 of 128 trials, but these data were still included in all analyses. We first analyzed behavioral responses to the targets and distractors. Accuracy was high across all conditions (OPA TMS, 73.1%; OPA control, 74.2%; OFA TMS, 69.0%, OFA control, 72.7%). An initial analysis of the behavioral data found no significant effect of normality (Shapiro-Wilks) across four groups (OFA and OPA, TMS and no TMS). A 2 × 2 mixed-design ANOVA found no main effect for Site (*F*_(1,22)_ = 1.56, *p* = 0.225, $\eta_{\text{p}}^{2}$ = 0.066) or TMS (*F*_(1,22)_ = 3.98, *p* = 0.058, $\eta_{\text{p}}^{2}$ = 0.153), and there was no interaction (*F*_(1,22)_ = 1.14, *p* = 0.298, $\eta_{\text{p}}^{2}$ = 0.049).

Next, we analyzed the eye movement data. In total, 14,961 fixations that started prior to the 1034 ms scene offset were exported for analysis. One-hundred and two of these fixations were removed for falling outside the scene image, as well as an additional 97 fixations and their subsequent saccades that preceded participants’ gaze landing outside of the scene image, leaving 14,762 fixations. These were broken up into 3312 and 3245 fixations in the first epoch (meaning the fixation started prior to the final TMS pulse at 400 ms after scene onset) for TMS and control conditions, respectively; and 4082 and 4123 fixations in the second epoch. Three different measures were then analyzed. Fixation position analysis used data from all 14,762 fixations. For the fixation duration (saccade latency) and saccade amplitude measures, 3024 fixations which overlapped scene offset were removed as subsequent saccades would be affected by the change in display, leaving 11,738 fixations and their ensuing saccades to analyze. These were broken up into 3307 and 3240 fixations in the first epoch for TMS and control conditions, respectively; and 2578 and 2613 fixations in the second epoch. There were six different analyses in total: 3 (temporal, retinotopic, spatiotopic) × 2 (horizontal, vertical saccades). Within each analysis, a mixed design ANOVA was used with Site (rOPA, rOFA) as the between subject factor, and Epoch (0–400 ms, 400–1034 ms) and TMS (trigger, control) as the within subject factors, creating a mixed 2 × 2 × 2 design. For all eight groups, within each measure, normality was checked with the Shapiro-Wilk test. All groups produced non-significant results unless stated.

### Saccade Latencies

**Figure 3:**
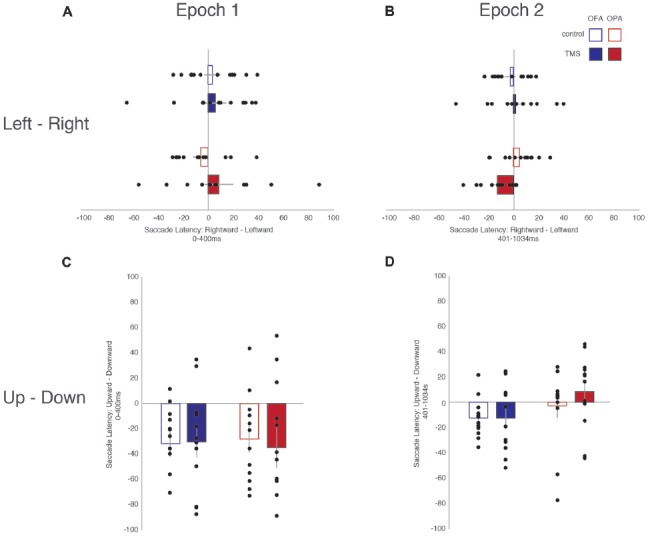
Differences in saccade latencies as a function of saccade direction and epoch. **(A)** Leftward—rightward saccade latencies during the first epoch (Epoch 1: 0–400 ms). **(B)** Leftward—rightward saccade latencies during the second epoch (Epoch 2: 401–1034 ms). **(C)** Upward—downward saccade latencies during the first epoch. **(D)** Upward—downward saccade latencies during the second epoch. Blue, OFA; red OPA. White boxes, control condition; filled boxes TMS condition.

All fixations were broken down into whether the following saccade was primarily horizontal or vertical (see “Materials and Methods” section). Within these divisions, left and rightward saccades were compared, as were upward and downward saccades (Table 1). For each participant, within each condition, the mean saccade latency in all four directions was determined. The mean latencies preceding leftward saccades were then subtracted from mean latencies preceding rightward saccades, and similarly those preceding downward saccades subtracted from upward saccades. Therefore, positive differences suggest that preparing a saccade rightward or upward takes longer; negative differences suggest that preparing a saccade to the left or downward takes longer (Figure 3).

**Table 1:** Saccade latency.

	OPA				OPA			
	Trigger Left	Trigger Right	Control Left	Control Right	Trigger Left	Trigger Right	Control Left	Control Right
0–400	206	212	210	213	206	215	211	205
StDev	41	35	22	15	48	64	36	44
401–1034	175	177	173	171	185	172	172	177
StDev	16	17	19	17	27	31	30	24
	**Trigger Down**	**Trigger Up**	**Control Down**	**Control Up**	**Trigger Down**	**Trigger Up**	**Control Down**	**Control Up**
0–400	214	184	223	191	216	181	221	193
StDev	40	46	14	34	64	62	48	53
401–1034	181	168	178	166	175	183	176	173
StDev	17	24	22	22	33	33	39	22

*Top Table. Mean and standard deviation of saccade latencies prior to leftward or rightward saccades in milliseconds. Bottom Table. Mean and standard deviation of saccade latencies prior to downward or upward saccades in milliseconds*.

For fixations preceding horizontal saccades, a mixed-design ANOVA found no main effect of Site (*F* < 1), Epoch (*F*_(1,22)_ = 1.02, *p* = 0.325, $\eta_{\text{p}}^{2}$ = 0.044), or TMS (*F* < 1). Likewise, there was no interaction of Site with either Epoch or TMS (*F*s < 1), and no significant interaction between Epoch and TMS (*F*_(1,22)_ = 3.92, *p* = 0.060, $\eta_{\text{p}}^{2}$ = 0.151). However, there was a significant three-way interaction between Site, Epoch and TMS (*F*_(1,22)_ = 5.15, *p* = 0.033, $\eta_{\text{p}}^{2}$ = 0.190).

In light of the three-way interaction, the data were split by Site and two follow-up two-way repeated measure ANOVAs were run. For the OFA group, there were no main effects or interaction of Epoch and TMS (*F*s < 1). For the OPA group, there was no main effect of Epoch or TMS (*F*s < 1), but there was a significant interaction (*F*_(1,11)_ = 9.91, *p* = 0.009, $\eta_{\text{p}}^{2}$ = 0.474). Paired sample *t*-tests then indicated there was no effect in the first epoch between the TMS and no TMS conditions, *t*_(11)_ = 1.72, *p* = 0.113 (8.6 and −5.8 ms, respectively), but that there was a significant difference in the second epoch, *t*_(11)_ = 2.50, *p* = 0.030 (−12.6 and 4.8 ms, respectively). The three-way interaction therefore appears to reflect an effect of TMS to OPA during the second epoch.

For fixations preceding vertical saccades, one of the eight groups produced a significant result in the Shapiro-Wilk test, which appeared to be a positive skew due to two participants in the OPA condition. When their results were removed the Shapiro-Wilk test was no longer significant; however, the overall pattern of statistical analyses remained the same regardless of their inclusion. A mixed-design ANOVA was therefore carried out with all 24 participants included. There was no main effect of Site (*F* < 1). There was a strong main effect of Epoch (*F*_(1,22)_ = 10.57, *p* = 0.004, $\eta_{\text{p}}^{2}$ = 0.324), with longer saccade latencies before downward saccades in the first epoch compared to the second (−31.2 and −4.9 ms, respectively). All other main effects and interactions were not significant (*F*s < 1).

Overall, these saccade latency results suggest an effect of TMS to OPA on horizontal, but not vertical saccades. This effect manifested in the second epoch, following the end of the TMS, with an increased saccade latency for leftward (contralateral) compared to rightward (ipsilateral) saccades.

### Saccade Amplitudes

**Figure 4:**
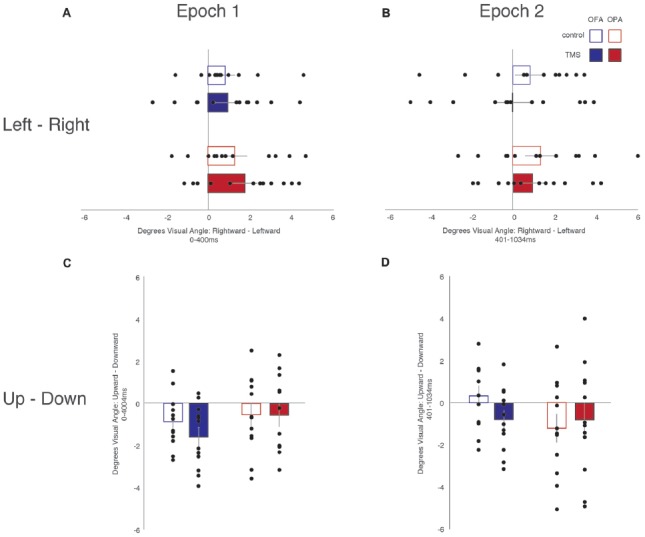
Saccade amplitude. **(A)** Leftward—rightward amplitude latencies during the first epoch. **(B)** Leftward—rightward saccade amplitude during the second epoch. **(C)** Upward—downward saccade latencies during the first epoch. **(D)** Upward—downward saccade latencies during the second epoch. Box colors are as in Figure 3.

Similar to the above analysis, saccades were broken down into whether they were leftward or rightward, or upward or downward (Table 2). For each participant, within each condition, the mean saccade amplitude in all four directions was calculated. Within each participant and condition, mean leftward saccade amplitudes were then subtracted from the mean of rightward saccade amplitudes, and similarly mean downward saccade amplitudes subtracted from the mean upward saccade amplitudes. Any positive differences suggest that rightward or upward saccades have greater amplitudes; negative differences suggest that leftward or downward saccades have greater amplitudes (Figure 4).

**Table 2:** Saccade amplitude.

	OPA				OPA			
	Trigger Left	Trigger Right	Control Left	Control Right	Trigger Left	Trigger Right	Control Left	Control Right
0–400	6.39	7.34	6.10	6.92	5.31	7.08	5.68	6.96
StDev	1.84	1.66	0.92	1.33	1.25	1.95	0.97	2.32
400–1034	9.69	9.67	9.29	10.11	8.70	9.61	9.06	10.38
StDev	2.14	1.94	2.01	2.01	1.90	2.62	2.20	2.56
	**Trigger Down**	**Trigger Up**	**Control Down**	**Control Up**	**Trigger Down**	**Trigger Up**	**Control Down**	**Control Up**
0–400	5.81	4.21	5.12	4.25	4.91	4.36	4.93	4.40
StDev	1.22	1.25	1.24	1.23	1.44	1.92	1.70	1.31
400–1034	6.99	6.23	6.71	7.04	7.48	6.70	7.70	6.49
StDev	1.29	0.77	1.17	1.24	2.74	1.99	2.36	1.24

*Top Table. Mean and standard deviation of saccade amplitudes prior to leftward or rightward saccades in degrees visual angle. Bottom Table. Mean and standard deviation of saccade amplitudes prior to downward or upward saccades in degrees visual angle*.

For horizontal saccades, a mixed-design ANOVA showed no main effect of Site (*F*_(1,22)_ = 1.10, *p* = 0.307, $\eta_{\text{p}}^{2}$ = 0.047), Epoch (*F*_(1,22)_ = 1.01, *p* = 0.325, $\eta_{\text{p}}^{2}$ = 0.044), or TMS (*F* < 1). Site did not interact with Epoch or TMS (*F*s < 1), Epoch did not interact with TMS (*F*_(1,22)_ = 2.57, *p* = 0.123, $\eta_{\text{p}}^{2}$ = 0.105), and there was no three-way interaction (*F* < 1).

For vertical saccades, a mixed-design ANOVA showed no main effect of Site, Epoch (*F*s < 1), or TMS (*F*_(1,22)_ = 2.32, *p* = 0.142, $\eta_{\text{p}}^{2}$ = 0.095). There was an interaction between TMS and Site (*F*_(1,22)_ = 5.86, *p* = 0.024, $\eta_{\text{p}}^{2}$ = 0.210), but not for Epoch and Site (*F*_(1,22)_ = 2.03, *p* = 0.168, $\eta_{\text{p}}^{2}$ = 0.085), Epoch and TMS, and no three-way interaction (*F*s < 1). The interaction between TMS and Site reflects larger downward saccades in the OFA TMS condition than the control condition (−1.18° and −0.27°, respectively; *p* = 0.019) while there was no difference between TMS and control in the OPA condition (−0.67° and −0.87°, respectively; *p* = 0.533).

Overall, these results suggest no effect of OPA TMS on saccade amplitude, although OFA TMS elicited larger downward saccades compared to the control condition.

### Fixation Position

Data was not broken down into horizontal and vertical saccades for this analysis. Instead, the x- and y-coordinates of each fixation were subtracted from the midline of the scene and converted to degrees of visual angle (Table 3). There was an overall left-to-right, top-to-bottom pattern across conditions (OPA and OFA; TMS and no TMS).

**Table 3:** Spatiotopic position.

	OFA		OPA		OFA		OPA	
	X Position Trigger	X Position Control	X Position Trigger	X Position Control	Y Position Trigger	Y Position Control	Y Position Trigger	Y Position Control
0–400	−0.86	−0.80	−0.67	−0.91	0.18	−0.14	−0.16	−0.06
StDev	1.18	0.92	0.94	0.94	0.80	0.91	0.52	0.43
400–1034	−0.08	0.35	0.64	−0.11	−0.66	−0.59	−0.25	−0.20
StDev	1.22	1.14	2.14	0.95	1.01	0.99	1.12	1.06

*Mean and standard deviation of fixation positions left and right of the midline (columns 1–4) and above and below the midline (columns 5–8), measured in visual angle*.

**Figure 5:**
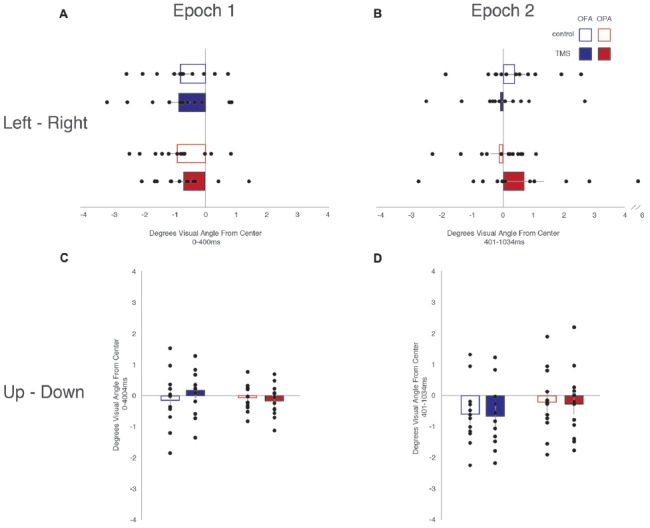
Fixation position from the vertical and horizontal midlines. **(A)** Leftward—rightward fixation position during the first epoch. **(B)** Leftward—rightward fixation position during the second epoch. **(C)** Upward—downward fixation position during the first epoch. **(D)** Upward—downward fixation position during the second epoch. Box colors are as in Figure 3.

When analyzing horizontal fixation position and distance from the vertical midline (x-coordinates; Figure 5), a mixed-design ANOVA showed no main effect of Site or TMS (*F*s < 1), but a main effect of Epoch (*F*_(1,22)_ = 11.53, *p* = 0.003, $\eta_{\text{p}}^{2}$ = 0.344), with fixations falling more to the left of the vertical midline in the first epoch and to the right of the midline in the second epoch (−0.81° and 0.20° from the midline, respectively). Additionally, while Epoch did not interact with either Site or TMS (*F*s < 1), there was an interaction between Site and TMS (*F*_(1,22)_ = 4.54, *p* = 0.045, $\eta_{\text{p}}^{2}$ = 0.171). Collapsing across epoch, fixations on OFA TMS trials landed further to the left than the control trials (−0.47° and −0.22°, respectively), while for OPA the opposite pattern was observed with TMS-trial fixations falling more to the right than the control trials (−0.02° and −0.51°, respectively).

When analyzing vertical fixation position and distance from the horizontal mid-line (y-coordinates; Figure 5), a mixed-design ANOVA showed no main effect of Site, TMS (*F*s < 1), or Epoch (*F*_(1,22)_ = 4.28, *p* = 0.051, $\eta_{\text{p}}^{2}$ = 0.163). Additionally, there was no interaction of Epoch and Site (*F*_(1,22)_ = 2.05, *p* = 0.166, $\eta_{\text{p}}^{2}$ = 0.085), Epoch and TMS (*F*_(1,22)_ = 1.96, *p* = 0.176, $\eta_{\text{p}}^{2}$ = 0.082), or TMS and Site (*F*_(1,22)_ = 1.69, *p* = 0.207, $\eta_{\text{p}}^{2}$ = 0.071). There was no three-way interaction (*F*_(1,22)_ = 3.19, *p* = 0.088, $\eta_{\text{p}}^{2}$ = 0.127).

Overall, these analyses of fixation position suggest that TMS to OPA biased fixation toward the ipsilateral visual field with no impact on vertical position.

## Discussion

In this study we investigated the role OPA plays in processing scene information. In particular, given OPA’s occipito-parietal location and retinotopic properties, we investigated whether OPA activity contributed to scene-related eye movements. To do this, we disrupted rOPA during scene viewing using TMS, and compared this to TMS of rOFA, which has a similar contralateral retinotopic representation but is preferentially activated during face viewing (Silson et al., 2015, 2016a). Overall, we found that stimulation of rOPA, but not rOFA, produces small but systematic effects on eye movements relating to the contralateral (left) visual field providing preliminary support for the idea that OPA processes scene information critical for eye movement guidance.

There was a general left-to-right, top-to-bottom gaze sweep of the scene images over the entire trial duration across all conditions (OFA and OPA; TMS and control), consistent with prior reports (also known as pseudoneglect, see Nuthmann and Matthias, 2014; Ossandón et al., 2014). On top of this left-to-right sweep, we found a spatiotopic effect whereby participants were more likely to fixate away from the left, contralateral, side of an image in the OPA TMS condition. Further, when examining saccade latencies, the time needed to program and execute a saccade, we found an effect during horizontal saccades in the second epoch of the rOPA group. This bias affected leftward saccades—heading into the disrupted visual field representation—increasing the latency to execute. Both of these effects were stronger in the second epoch, after the pulse train finished, although the interaction with epoch was only significant for saccade latency. The delayed gaze bias suggests that disruption of OPA activity may stall the accrual of information needed for executing future saccades to new scene locations, rather than information needed for the immediate saccade. Together these findings suggest that OPA processes scene information, which is then utilized by the eye movement network leading to changes in the oculomotor system.

In addition to the contralateral bias, Silson et al. (2015) found a comparatively weaker, lower-visual field bias for OPA, suggesting that TMS might also affect elevation. However, the bias for our individual stimulation sites (Figure 2) was not as strong as previously reported for the whole functional regions-of-interest. When comparing eye movements directed upwards and downwards, we found a large saccade latency delay prior to making downward saccades that did not interact with site, TMS or Epoch. This downward directional delay is common in free viewing (Tatler and Vincent, 2008) and may have been large enough to wipe out potentially smaller effects of TMS. There was a spatial bias found with OFA TMS trials with larger downward saccades compared with control trials, while there was minimal difference between the OPA TMS and control conditions. However, this effect appears to be due to OFA control trials not following the same downward trend as the other three conditions (OFA trigger, OPA trigger, OPA control; see Figure 4). This may reflect an anticipatory effect by the OFA group, however we did not collect responses regarding potential strategies during the task, so this cannot be formally tested here.

Our combined results suggest that activity within OPA, a region dedicated to visual analysis of scenes, affects saccade processing during free-viewing of real-world scenes, particularly referencing information in the contralateral visual field. When activity in this region was disrupted, we saw evidence of gaze bias toward the ipsilateral visual field. Previous findings suggest OPA demonstrates preferential activity for local scene information (Kamps et al., 2016a) and contributes to encoding navigable space (Dilks et al., 2011; Julian et al., 2016; Bonner and Epstein, 2017). In keeping with its occipito-parietal position, our findings additionally suggest that OPA might play a causal role in analyzing local scene information leading to effective eye movement guidance. Eye movements are vital for a range of real-world tasks such as search and encoding, and fixation positions rely on parsing a variety of information (Nuthmann, 2014; Malcolm et al., 2016; Nuthmann and Malcolm, 2016; Henderson, 2017). Whether the information that affects eye movements is preferentially linked to a specific function (e.g., discerning navigability, searching for task-relevant items) or a particular set of features in contralateral space remains an open question for follow-up research.

The size of the effects we report are relatively small compared with studies using TMS to disrupt cortical sites more directly involved with the oculomotor system. For instance, when a 10 Hz five-pulse train similar to ours was delivered to the frontal eye field (FEF), it produced a 51 ms increase in saccade latency during a prosaccade task (Taylor et al., 2006). Similarly, a single-pulse given to FEF 200 ms after a “go” signal found a 37 ms increase in mean saccade latency during prosaccades to the contralateral visual field compared to a no TMS condition (Nagel et al., 2008). Yang and Kapoula (2011) also found that a single pulse to FEF, delivered 100 ms after a “go” signal, increased memory guided saccade and convergent/divergent latencies by 21–56 ms. Conversely, in a study targeting the posterior parietal cortex (PPC), TMS disruption produced a ~30 ms increase in saccade latencies in a gap paradigm (Kapoula et al., 2001).

By comparison, the results found in the current study are relatively smaller (e.g., a 13 ms increase in saccade latency toward the contralateral compared to the ipsilateral visual field, in the epoch after the pulse-train had ceased). This could be due to a number of reasons. First, unlike FEF and PPC, OPA is not considered part of the oculomotor system (Awh et al., 2006). Rather than directly affecting eye movements, we are suggesting that activity within OPA provides information to the oculomotor system which may be used depending on the viewer’s needs. This may explain why we found stronger effects in the second epoch, after the last pulse. OPA analyses visual information; during disruption the oculomotor system may already be dealing with an existing saccade program, before the disrupted information from OPA has been received (although this would need to be formally tested). Second, most studies utilizing TMS disruption to measure effects on saccadic activity use tightly controlled paradigms, presenting single targets to fixate at a specified time, restricting possible saccade outcomes. Even visual search studies tend to use simple arrays of discrete items, that required only a single saccade to a target (e.g., Juan et al., 2008; Muggleton et al., 2011). In the present study, participants freely viewed scenes with multiple potential points of fixation; a disrupted region of space may therefore have less influence when there are other competing points of interest. Third, most previous studies use a range of time-locked pulses or double-pulses to locate the timing of peak activity. Here, we provided a five-pulse train over 500 ms at 10 Hz. While the first pulse was time-locked to scene onset, the remaining four pulses could occur during any stage of a fixation or saccade.

Our study suggests that in combination, we can probe the behavioral consequences of cortical activation with high spatial and temporal resolution. However, there are important limitations of our study. First, while we demonstrate TMS site-specificity for our effects (OPA vs. OFA), it could be argued that stimulation of any region of dorsal visual cortex would elicit similar effects to those we observed. Our results show that stimulation of a scene-selective, but not face-selective representation of the contralateral visual field biases gaze allocation during scene viewing. Thus, OFA serves as a control to show that our findings do not simply reflect an effect of stimulation on any category-selective visual region representing that part of visual space. But OFA is presumably farther from the dorsal visual pathway than OPA and may be more associated with the ventral pathway. Future work will need to establish the spatial specificity of the effects we observed in more detail.

Second, we have not demonstrated the effects we observed are specific to scenes. Ideally the study would have contrasted stimuli as well as site. For example, demonstrating that TMS to OPA causes an ipsilateral bias in scene images whereas TMS to OFA causes a similar effect when viewing face images would have strengthened the interpretation of the results. However, the dissimilarities between scene and face stimuli—shape, perceptual cues, depth, etc — would make reliably comparing eye movement data across the two stimulus sets difficult. More generally, creating stimuli that maintain the size, shape and low-level features of a scene, while also not changing the pattern of eye movements during free-viewing or indeed the viewers’ task, is not trivial. However, as OPA is not part of the established oculomotor system it is unlikely that disruption would directly affect gaze independently of what was being viewed. Nevertheless, these are important questions for future work.

Third, it is not possible from the current results to know when OPA activity affects gaze, although the stronger results in the second epoch suggests that activity is relevant for planning future saccades beyond the next immediate saccade. The relevance of OPA activity could be over two different epochs. The first is within the epoch of a single fixation. The function of gaze varies over the course of a fixation between processing information at the fovea and processing information in the periphery (Rayner, 1998; van Diepen et al., 1998). Future research could apply a single- or double-step pulse time-locked to fixation durations, and record sensitive eye movement measures to isolate when peak disruption for a cortical region occurs (see, Koivisto et al., 2011; Camprodon et al., 2012; Pitcher et al., 2012). The second epoch stretches over the duration a scene is visible, as the nature of scene processing varies from time since onset (Fei-Fei et al., 2007; Kadar and Ben-Shahar, 2012; Malcolm et al., 2014). OPA activity may affect gaze depending how long a scene image has been visible, in which case time-locking a pulse-train to varying delays after onset may likewise produce different gaze behaviors. In these ways, future research can begin to complement the present findings to specify the critical time-points from which OPA activity biases gaze allocation during scene viewing.

Finally, OPA is active during the processing of dynamic scenes (Çukur et al., 2016), and may even show more activity for dynamic than static scenes (Kamps et al., 2016b). However, it is uncertain how our results would extend to dynamic scenes. Gaze allocation in dynamic scenes will be driven by motion cues (as well as feature cues), which are likely processed in other areas such as MT and/or V3A. Thus, to the extent that gaze allocation is driven by such motion cues, TMS to OPA may be expected to have less impact. These considerations suggest that an interesting future study might be to compare the effect of TMS to OPA and MT/V3A on static and dynamic scenes, respectively.

In summary, we show that selective disruption of rOPA, but not rOFA, produces small but systematic effects on eye movement patterns with respect to the contralateral visual field. Collectively, these data provide preliminary evidence that OPA plays a causal role in processing scene information critical for eye movement guidance.

## Author Contributions

GLM helped conceive the project idea, collect stimuli, run participants, analyze data and writing of the manuscript. EHS helped conceive the project idea, acquire and analyze neuroimaging data, run participants and writing of the manuscript. JRH helped collect stimuli, run participants and writing the manuscript. CIB helped conceive the project idea, analyze data and writing of the manuscript.

## Conflict of Interest Statement

The authors declare that the research was conducted in the absence of any commercial or financial relationships that could be construed as a potential conflict of interest.

## Footnotes

^1^http://afni.nimh.nih.gov/afni
